# Great balls of fire: activation and signalling of inflammatory caspases

**DOI:** 10.1042/BST20200986

**Published:** 2021-06-01

**Authors:** Georgia Bateman, Benjamin Hill, Ryan Knight, Dave Boucher

**Affiliations:** 1 York Biomedical Research Institute, University of York, Heslington, York, U.K.; 2 Department of Biology, University of York, Heslington, York, U.K.

**Keywords:** caspase, inflammasome, innate immunity, protease signalling

## Abstract

Innate immune responses are tightly regulated by various pathways to control infections and maintain homeostasis. One of these pathways, the inflammasome pathway, activates a family of cysteine proteases called inflammatory caspases. They orchestrate an immune response by cleaving specific cellular substrates. Canonical inflammasomes activate caspase-1, whereas non-canonical inflammasomes activate caspase-4 and -5 in humans and caspase-11 in mice. Caspases are highly specific enzymes that select their substrates through diverse mechanisms. During inflammation, caspase activity is responsible for the secretion of inflammatory cytokines and the execution of a form of lytic and inflammatory cell death called pyroptosis. This review aims to bring together our current knowledge of the biochemical processes behind inflammatory caspase activation, substrate specificity, and substrate signalling.

## Introduction

Proteases are central enzymes that mediate numerous signalling roles to ensure cellular functions and organismal homeostasis [1]. Discovered more than 20 years ago, caspases are key signalling proteases that control various cell death processes and have been linked to inflammation and non-cell death-related functions [2–5].

Inflammatory caspases are a caspase subset activated by cellular platforms called inflammasomes [6–8]. Albeit mediating inflammasome signalling, our understanding of the biochemistry and the cellular processes governed by inflammatory caspases is limited. This mini-review aims to bring together our understanding of the mechanisms regulating inflammatory caspases activation, signalling and regulation.

## Caspases…what's in the name?

The term caspase [2,9] is derived from the cysteine catalytic site used by the protease, and its rare specificity for cleavage at the carboxy-terminal side of Aspartic acid residues (D); **c**ysteine-dependent **asp**artate-specific prote**ases**. Caspases use a catalytic dyad composed of an histidine (H237 in caspase-1) and a cysteine (Cys285 in caspase-1) [2]. Initially discovered in *Caenorhabditis elegans (C.elegans)* [3,5], the role of caspases in development and innate immunity have since been characterised in a wide range of multicellular organisms. Recent work also clarifies important functions of caspases outside these processes, including proliferation, migration, and differentiation [10–13].

**Figure 1.**
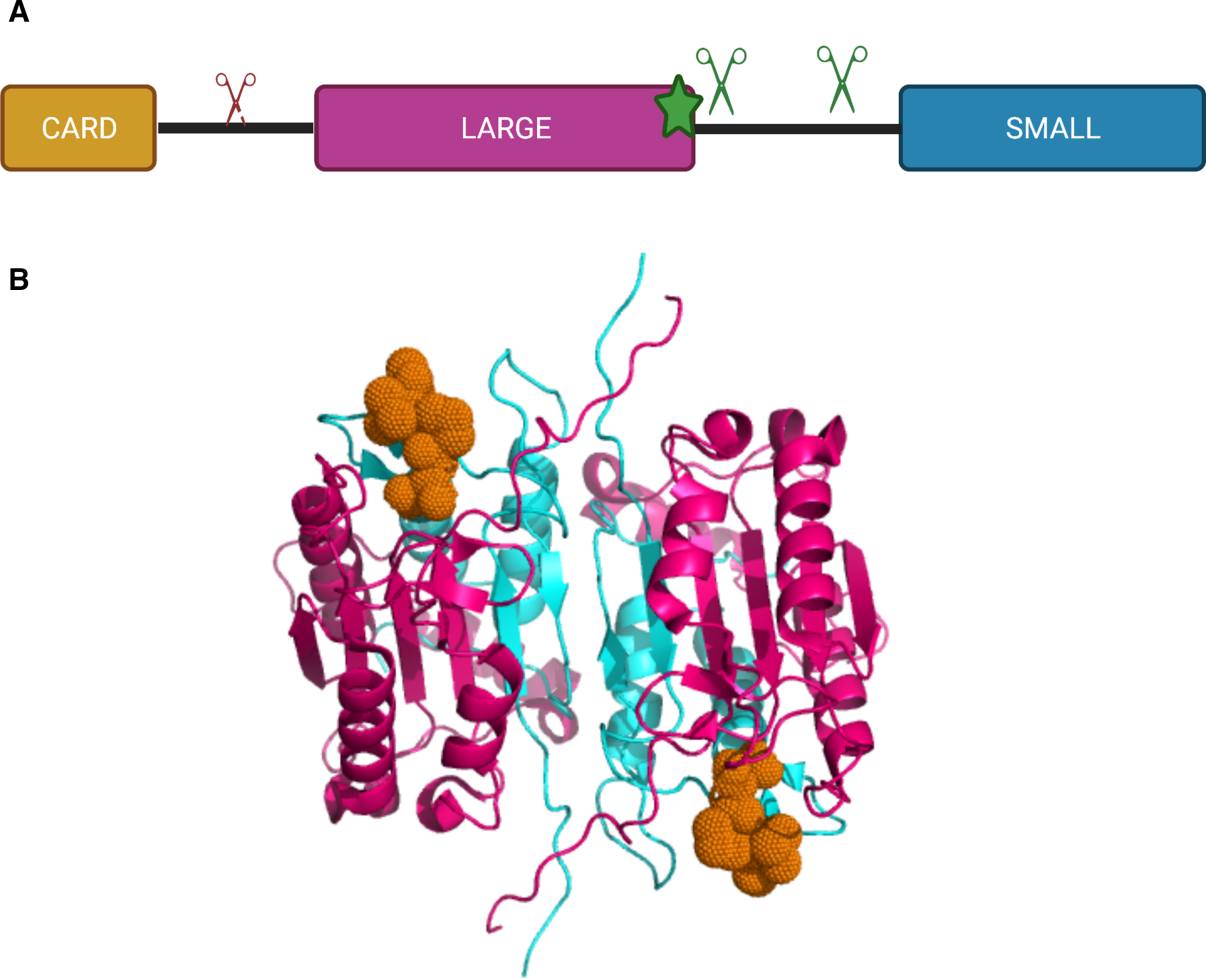
Structural organisation of an inflammatory caspase. (**A**) Inflammatory caspases are composed of a CARD domain and a catalytic subunit, divided into a large and a small subunit. Caspases use a catalytic cysteine (shown as a star) to support their catalytic activity. Scissors represent inhibitory (red) and activating (green) self-processing sites. (**B**) Crystal structure of caspase-1 bound to the active-site inhibitor VX765 (PDB: 6PZP). The large subunit (pink) and the small subunit (cyan) of a caspase-1 dimer interacting with the active site inhibitor VX-765 (orange).

Caspases have a conserved modular organisation: a N-terminal domain (of variable length and function), a large catalytic subunit, and a small catalytic subunit [2] (Figure 1). These domains are separated by flexible linkers sensitive to proteolysis, the interdomain linker (IDL) and the recruitment domain linker (RDL). To date, twelve caspases have been identified in humans and ten in mice [2]. A classification system for caspases was developed, dividing each caspase into two main groups in accordance with their function, structure, and activation mechanism (Table 1).

**Table 1:** Human caspases functions and activation mechanism overview

Caspase	Function/activation mechanism
1	Inflammation/dimerisation
2	Apoptosis/dimerisation
3	Apoptosis (Executioner)/dimerisation
4	Inflammation/dimerisation
5	Inflammation/dimerisation
6	Apoptosis (Executioner)/cleavage
7	Apoptosis (Executioner)/cleavage
8	Apoptosis (Initiator)/dimerisation
9	Apoptosis (Initiator)/dimerisation
10	Apoptosis (Initiator)/dimerisation
12	Unclear, catalytically inactive
14	Keratinocyte differentiation/dimerisation

The first caspase group, the apoptotic caspases, can be further subdivided into initiator and executioner caspases based on their role within the apoptotic pathway. The initiator caspases (caspase-8, -9, and -10), are monomeric and contain a long homotypic N-terminal domain required for recruitment to their respective activation platform. The initiator caspases can then be further divided into whether they participate in the extrinsic or intrinsic apoptotic pathway. The extrinsic pathway involves caspase-8 and -10, which are activated by complexes like the death-inducing signalling complex (DISC) following binding of death ligands to their cognate receptors [14,15]. The intrinsic apoptotic pathway involves caspase-9, activated by the apoptosome following sensing of various intracellular signal like DNA-damage [16,17]. Once activated, the initiator caspases activate the executioner apoptotic caspases (caspase-3, -6 and -7) by cleaving their IDL [2,18].

Executioner caspases contain a short pro-domain (<30 residues) and are synthesised as inactive dimeric zymogens. Cleavage of executioner caspases by the initiator caspases allows full activation and therefore the cleavage of specific substrates to execute apoptotic cell death [19].

The second main caspase group is the inflammatory caspases [20]. The inflammatory caspases are encoded by three genes in humans (CASP1, CASP4, CASP5), and two in mice (casp1, casp11), clustered on a single locus, chromosome 11 in humans, and on chromosome 9 in mice. In mice, caspase-12 is also considered an inflammatory caspase [21] but roles of caspase-12 in inflammasome signalling have been debated [22]. Humans express a truncated and inactive version of caspase-12 [23,24], therefore this caspase will not be discussed further. The best characterised inflammatory caspase is caspase-1. This caspase, originally named IL-1β converting enzyme (ICE), was identified whilst studying the protease involved in the processing of the proIL-1β cytokine [4,25–27]. Shortly after, caspase-4, -5 and -11 [28] were linked to cell death and endotoxin responses. Caspase-1 is activated by a signalling complex called the canonical inflammasome, whereas caspase-4 and -11 (and potentially -5) are activated by the non-canonical inflammasome. Caspase-1 and caspase-4 are constitutively expressed in most cell types whereas caspase-5 expression is interferon inducible [29]. A recent study identified a gain-of-function mutation in CCAAT enhancer–binding protein epsilon (CEBPE) causes an autoinflammatory inflammasomopathy that leads to constitutive caspase-5 expression [30].

A few caspases fall out of the traditional classification due to their unique functions. An example of this is caspase-14, a caspase involved in keratinocyte differentiation [31]. Caspase-2 is another caspase member that has been linked with apoptotic processes and with innate immune functions, however does not comfortably fit into the current classification system [32].

The remainder of this review will focus on the mechanisms that govern inflammatory caspase activity.

## Inflammatory caspase activation

### Caspase-1

Inflammatory caspases exist as monomers under the cellular resting state and require dimerisation to become active. This dimerisation step is tightly regulated and is mediated by large, multi-protein signalling platforms called inflammasomes [6]. Inflammasomes are composed of a pattern recognition receptor (PRR) which senses danger-associated molecular patterns (DAMPs) or pathogen-associated molecular patterns (PAMPs), an adaptor protein (Apoptosis-associated speck-like protein (ASC)) and an inflammatory caspase [7,8].

PRR-activating inflammasomes are diverse in nature and recognise or respond to a multitude of chemically different ligands (summarised in Table 2).

**Table 2:** Inflammasome-forming pattern recognition receptors

Pattern recognition receptor	Examples of activating stimuli
NLRP1	Proteases from pathogens [39,40]
	viral dsRNA [41]
	Toxoplasma gondii [42]
NLRP3	Pore-forming toxins (Hemolysin, Candidalysin) [43,44]
	B-glucan [45]
	ATP [46]
	Urate crystal [47]
NLRP6	Lipoteichoic acid (LTA) [48]
NLRP7	Lipopeptide [49]
NLRC4/NAIP	T3SS proteins, Flagellin [50]
AIM2	Cytosolic DNA [51,52]
Pyrin	Rho GTPase inactivation [53]
GBPs	LPS [54–56]

Inflammasome-activating PRRs contain either a Pyrin domain (PYD) or Caspase activation and recruitment domain (CARD), both of which belong to the death-fold domain family [33]. The presence of these homotypic domains is a unifying feature of inflammasome-activating PRRs, which can therefore be divided into PYD-containing (NLRP3, NLRP6, NLRP7, AIM2, Pyrin) or CARD-containing (NLRP1, NLRC4, CARD8) PRR. Following PRR activation and oligomerisation, these domains undergo homotypic domain–domain interactions (PYD–PYD or CARD–CARD) that allow the recruitment of the adaptor protein ASC. ASC is a 22 kD adaptor protein, containing both a PYD and CARD domain. ASC–PYD oligomerisation leads to the formation of ASC filaments, and interactions between these filaments through ASC–CARD leads to the formation of the ASC speck, with a single cellular focus of ∼1 µm [34,35]. The ASC speck recruits caspase-1 monomers through CARD–CARD homotypic interactions, increasing local caspase-1 concentration therefore promoting caspase-1 dimerisation and allowing its activation [36]. Caspase dimerisation occurs through an interface located in the small catalytic subunit [2]. Dimerisation of caspase-1 induces basal activity and allows for the processing of the interdomain linker (IDL), which leads to structural reorganisation and stabilises the active site to generate a fully active caspase-1 species called p33/p10 (Figure 1) [36]. This activation mechanism, shared with initiator caspases, is known as proximity-induced dimerisation [19]. The caspase-1 p33/p10 species has the ability to process its established substrates (IL-1β, IL-18 and GSDMD) to mediate cell death and cytokine secretion (Figure 3). Caspase-1 subsequently cleaves its RDL to generate the p20/p10 species and dissociate from the inflammasome and become inactive [36]. Dimeric full-length caspase-1 is also partially active and can mediate cell death, but not cytokines processing [37].

The ASC speck can also recruit and activate caspase-8 to trigger apoptosis [38].

### Caspase-4 and -5

Inflammatory caspase-4 and -5 in humans, and caspase-11 in mice, are activated by the non-canonical inflammasome. Caspase-5 is conserved only in a few species (humans and great apes) and is believed to be the consequence of a genetic duplication. Until recently, these caspases were believed to directly binding lipopolysaccharide to trigger direct caspase dimerisation and activation [57]. However, recent studies identified cellular factors that facilitate the presentation of hydrophobic bacterial LPS to these caspases. Interferon-inducible guanylate binding proteins (GBPs) [58] recognise the outer section of LPS on cytosolic bacteria and allow for the assembly of an inflammasome directly on the bacteria. In human epithelial cells, this assembly platform is composed of GBP1, 2, 3 and 4 [54–56,59]. Outer membrane vesicles [60] can also activate the non-canonical inflammasome in a GBP-dependant manner [61]. In mice, GBPs facilitate the recruitment and localisation of IRGB10 to the membrane of invading pathogens, resulting in the destruction of the pathogen membrane and subsequent release of LPS and DNA, activating the non-canonical and AIM2 inflammasomes, respectively [62,63]. Humans do not express a functional orthologue of IRGB10. Caspase-4 and -5 active species are yet to be fully characterised; however, studies suggest that the p32/p10 form of caspase-4 could be the active species [64]. Studies into caspase-11 have supported this, showing that caspase-11 needs to be cleaved at the IDL to generate a fully active species [65,66]. Caspase-4 and -11 have also been suggested to be activated by the NLRP6 inflammasome downstream of Lipoteichoic acid (LTA) recognition [48]. However, the molecular basis of this process is not fully understood. Fatty acids and oxidised lipids have also been suggested to be endogenous ligands for the non-canonical inflammasome with cell-specific outcomes [67–69].

The cellular context controlling caspase-5 activation remain elusive. Specific LPS structures (e.g. Outer membrane vesicles from *Pseudomonas aeruginosa*) [70] and NLRP1 [6] were suggested to activate caspase-5. However, features enabling caspase-5 activation (instead of caspase-4) are subject of current investigations.

## Caspase specificity

**Figure 2.**
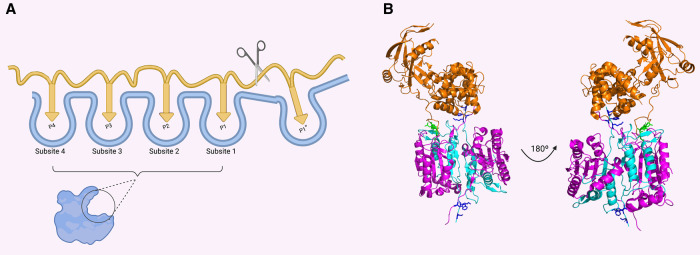
Substrate recognition mechanism by caspases. (**A**) Representation of the caspase substrate-binding pocket. Four subsites on the caspase (Subsite 4 to Subsite 1) recognise the classical tetrapeptide on the substrate (P4 to P1). (**B**) Structure of the complex between caspase-11 (pink and cyan) and Gasdermin D (PDB: 6VIE) demonstrates that caspases can use exosite to recognise specific substrates. The structure shows the tetrapeptide recognise by the substrate-binding pocket (green) and the exosite (blue) that binds an additional region on Gasdermin D.

Inflammatory caspases are highly specific proteases that cleave defined protein substrates to orchestrate the innate immune responses. In the following section, we will discuss how caspases achieve this specificity through diverse mechanisms (Figure 2).

**Figure 3.**
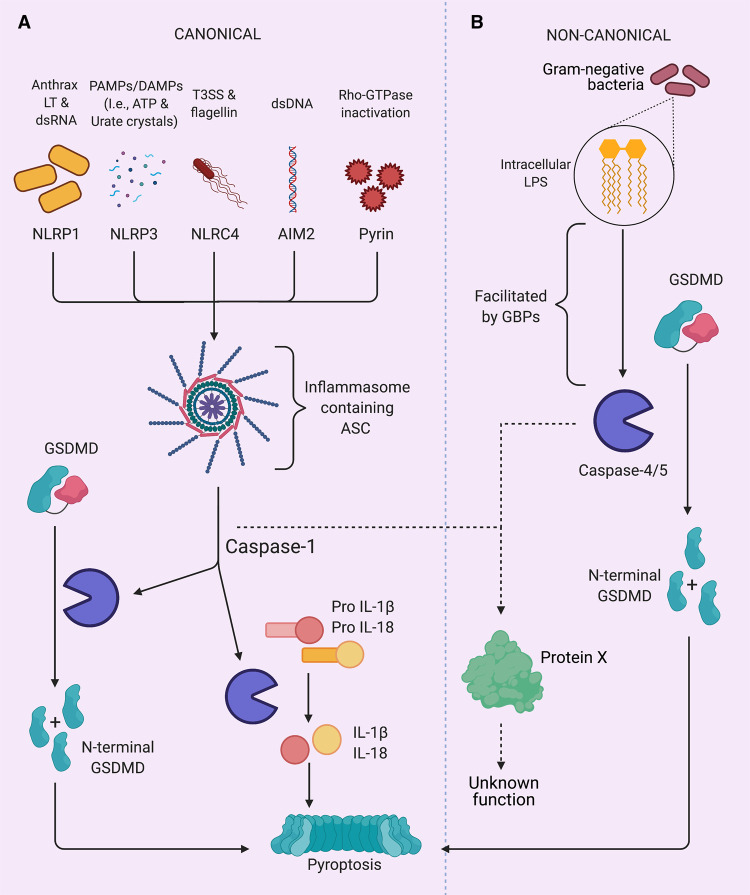
Canonical vs non-canonical inflammatory caspases signalling. (**A**) PRRs recognise recognise DAMPs and PAMPs to activate the canonical inflammasome. This results in the activation of caspase-1 which cleaves GSDMD leading to pore formation and pyroptosis. Caspase-1 also activates pro-inflammatory cytokines IL-18 and IL-1b, which are release during pyroptosis and other substrates with unknown functional consequences (protein X). (**B**) Intracellular LPS from Gram negative bacteria activates the non-canonical inflammasome. GBPs aid the activation of caspase-4 and -5 by LPS. Caspase-4 and -5 cleave GSDMD, inducing pyroptosis, and other substrates with unknown functional consequences (protein X).

### Primary specificity

Caspases natural substrate must be present in the same cellular compartment as the caspase and their cleavage site present specific features. First, the P1′ position is usually occupied by a small aliphatic amino acid. Secondly, the cleavage site is usually located in a solvent-exposed flexible structural element and is accessible to the protease active site. Finally, caspase substrate often displays an optimal primary cleavage sequence [71].

The substrates primary cleavage sequence bind through the caspase substrate-binding pocket, which can generally accommodate four amino acids from the substrate. According to the Schechter-Berger nomenclature [72], caspases recognise an aspartate in position P1 and several different amino acids in position P4 to P2 (Figure 2). Using small peptide library, the preferred substrate sequence for each caspase has been defined. Inflammatory caspases prefer aromatic or hydrophobic amino acids in position P4, glutamic acids in position P3, and small aliphatic amino acids in position P2 [73].

Recently, caspases were shown to cleave artificial substrates containing glutamic acid and phosphorylated serine in position P1, expanding the potential sequence of substrates [74].

Although the focus has been towards the primary tetrapeptide, multiple studies have highlighted the influence of extended subsites on caspase specificity. The Salvesen group identified extended subsites in caspase-11 and caspase-5 substrates that increase cleavage of selected substrates [75,76]. Extended subsites have also been suggested in other caspases [32].

The ability of certain caspases to cleave these primary sequences can also be influenced by post-translational modifications of the substrates. For example, phosphorylation of the substrates primary tetrapeptide has been shown to influence the ability of apoptotic caspases to cleave certain substrates [74,77]. However, its influence on inflammatory caspase specificity is currently unclear.

Caspase specificity to small peptides led to the development of caspase inhibitors. However, these inhibitors display relative specificity as they can target other caspases [78]. For example, an inhibitor derived from caspase-8 favourite recognition sequence will also inhibit efficiently other caspases [78].

Determinants outside the substrate-binding pocket have also been shown to influence caspase specificity [79].

### Exosites

Exosites are structural motifs that allow binding of substrates independently of the primary substrate binding pocket. Although insufficient to allow substrate cleavage on their own, exosites enhance the cleavage efficiency of specific substrates and are used by various proteases to achieve protease specificity.

Exosites were first observed in apoptotic caspases. Caspase-7 harbours an exosite in its N-terminal domain that consist of four lysines [79]. Recently, this exosite has been suggested to bind RNA, facilitating RNA-binding protein cleavage by caspase-7 [80]. Similar sequences have been found in other apoptotic caspases, such as caspase-6 [81].

Until recently, no structure of caspases with their protein substrates were available. However, two recent papers outline unprecedent details on how exosites allow substrate specificity by inflammatory caspases.

Shao's and Tsiao's lab reveal the structure of caspase-1, -4 and -11 bound to the C-terminal fragment of GSDMD [82] or the full-length GSDMD [83]. Their structures reveal that the interaction between the loop L2 and L2’ of the caspase IDL creates a new binding site for the C-terminal domain of GSDMD, increasing caspase affinity for GSDMD and reducing the impact of a defined primary sequence (positions P4–P1 according to Schechter–Berger nomenclature [72]) for GSDMD to be cleaved by inflammatory caspases (Figure 2).

So far, GSDMD is the only inflammatory caspases substrate that has been clearly shown to bind on an inflammatory caspase exosite. However, recent evidence suggests that similar sites are also present for IL-1β [84].

Developing inhibitors that target exosites instead of the primary substrate-binding pocket bear the promise of more specific inhibitors.

## Inflammatory caspases substrates and signalling

Caspase substrate cleavage has three functional consequences: a loss-of-functions, a gain of functions or a no-consequence effect. Caspase cleavage may also affect protein stability and target specific substrates to proteasomal degradation [85,86].

Proteomic studies have identified substrates for caspase-1 in mice [87,88] and humans [89] but so far failed to successfully identify caspase-4 and-5 substrates. Table 3 summarise substrates that have been identified by forward and reverse proteomics and shows that caspases are involved in multiple pathways (Figure 3).

**Table 3:** Inflammatory caspases substrates

Substrate	Uniprot ID	Cleavage site (P4–P1′)	Function	Caspase	Gain/Loss	Conserved	Reference
IL-1a	P01583	Ile/Ala/Asn/**Asp(104)**/Ser	Pro-inflammatory cytokine	5	G	M/H	[95]
IL-1B	P01584	Phe/Glu/Ala/**Asp(27)**/Gly	Pro-inflammatory cytokine	1/5	G	D/M/H	[4,89]
IL1B	P01584	Tyr/Val/His/**Asp(116)**/Ala	Pro-inflammatory cytokine	1	G	D/M/H	[4,89]
IL-18	O95256	Leu/Glu/Ser/**Asp(36)**/Tyr	Pro-inflammatory cytokine	1/4/5	G	M/H	[75,111]
IL-37	Q9NZH6	Trp/Glu/Lys/**Asp(20)**/Glu	Anti-inflammatory cytokine	1	G	H	[94]
casp3	P42574	Ile/Gly/Thr/**Asp(175)**/Ser	Apoptotic pathway	1/4	G	M/H	[112,113]
casp7	P55210	Ile/Gln/Ala/**Asp(198)**/Ser	Apoptotic pathway	1	G	D/M/H	[87]
Bid	P55957	Ile/Glu/ala/**Asp(75)**/Ser	Apoptotic pathway	1	G	D/M/H	[107,114]
BAP31	P51572	Ala/Ala/Val/**Asp(231)**/Gly	Apoptotic pathway	1	G	D/H	[115]
PARP1	P09874	Asp/Glu/Val/**Asp(214)**/Gly	Apoptotic pathway	1	L	D/M/H	[116]
SYAP1	Q96A49	Phe/Val/Ser/**Asp(278)**/Ala	Signal Transduction	1/5	UN	D/M/H	[89]
GSDMD	P57764	Phe/Leu/Thr/**Asp(275)/**Gly	Pyroptosis	1/4/5	G	D/M/H	[89,97,99]
Actin	P60709	Leu/Val/Val/**Asp(11)**/Asn	Cell structure	1	UN	D/M/H	[89]
Actin	P60709	Gly/Gln/Lys/**Asp(51)**/Ser	Cell structure	4	UN	D/M/H	[89]
Actin	P60709	Asp/Ser/Gly/**Asp(157)**/Gly	Cell structure	1	UN	D/M/H	[89]
Gelsolin	P06396	Asp/Glu/Thr/**Asp(403)**/Gly	Cell structure	1	G	M/H	[89]
Spectrin	Q13813	Asp/Glut/Thr/**Asp(1185)**/Ser	Cell structure	4	UN	D/M/H	[117]
ARPC5	O15511	Asp/Glu/Glu/**Asp(29)/**Gly	Cell structure	1	UN	D/M/H	[89]
RCSD1	Q6JBY9	Glu/Glu/Val/**Asp(272)**/Gly	Cell structure	1	UN	H	[89]
IQGAP1	IQGAP1	Asp/Glu/Val/**Asp(8)**/Gly	Cell structure	1	UN	D/M/H	[89]
GAPDH	P04075	Lys/Thr/Val/**Asp(189)**/Gly	Metabolism	1	L	D/M/H	[88]
ENO1	P06733	?	Metabolism	1	UN	H	[88]
TPI1	P60174	?	Metabolism	1	UN	H	[88]
PIP4K2B	Q9UBF8	Phe/Ser/Val/**Asp(488)**/Ser	Kinase	1	UN	D/M/H	[89]
BASP1	P80723	Thr/Lys/Ser/**Asp(165)**/Gly	Channel	1	UN	H	[89]
CALU	O43852	Tyr/Ile/Gly/**Asp(216)**/Met	Calcium-binding	1	UN	D/M/H	[89]
USP10	Q14694	Leu/Glu/Asn/**Asp(138)**/Gly	Ubiquitin protease	1	UN	D/M/H	[89]
HOIP	Q96EP0	Leu/Glu/Pro/**Asp(348)**/Leu	Ubiquitin ligase	1	L	M/H	[118]
HOIP	Q96EP0	Leu/Val/Val/Val/**Asp(387)**/Ser	Ubiquitin ligase	1	L	D/M/H	[118]
TFAP2A	P05549	Asp/Arg/His/**Asp(19)**/Gly	Transcription	1	UN	M/H	[119]
Max	P61244	Ile/Glu/Val/**Glu(10)**/Ser	Transcription	5	UN	M/H	[110]

### Cytokines

Caspase-1 was originally identified as an Interleukine-1β (IL-1β)-converting enzyme and was originally characterised to cleave and mature this cytokine. Caspase-1 also processes the IL-18 pro-form into its mature form. The cytokines IL-1β and IL-18 are unconventionally secreted through GSDMD pores or following cell lysis [90,91]. Caspase-4 also cleave these cytokines, although much less efficiently than caspase-1 [75]. Cleavage of IL-1β and IL-18 leads to the recruitment of additional phagocytes and contributes to the generation of a fever. Caspase-8 also cleaves IL-1β and IL-18 during multiple situations [92,93].

Caspase-1 also cleaves IL-37, an anti-inflammatory cytokine, to promote IL-37 nuclear translocation and genetic repression of anti-inflammatory cytokines [94].

Caspase-5 has been reported to cleave IL-1α in senescent cells, a process that may contribute to aging-associated inflammation [95].

### Gasdermin D

Gasdermin D (GSDMD) is a central executor of pyroptosis (Figure 3). Following cleavage by caspase-1, -4 (-11), -5 and -8, the GSDMD N-terminal fragment is freed from its inhibitory counterpart (C-terminal fragment) and is able to form pores at the plasma membrane and into different organelles [96–99]. GSDMD pores allow secretion of pro-inflammatory cytokines (IL-1β, IL-18) and the release of a myriad of DAMPS (e.g. ATP, Galectin-1) [100,101]. Additionally, GSDMD pores generate potassium efflux to allow caspase-1 activation through the NLRP3 inflammasome, downstream of the non-canonical inflammasome [102,103]. Terminal membrane rupture downstream of GSDMD pores has been shown to be mediated by the membrane protein NINJ1 [104]. In neutrophils, GSDMD cleavage by non-canonical inflammatory caspases allows the generation of neutrophil extracellular traps [105].

### Other substrates

Caspase-1 can cleave proapoptotic proteins like Bid and caspase-3 and -7. It has been suggested to control infection by specific pathogens and stands as a backup cell death mechanism if pyroptosis is counteracted by pathogens [106–108].

Caspase-1, -4, -5, and -11 can cleave and inactivate cGAS to control type I IFN response and modulate antiviral responses [109].

Caspase-1 has been shown to cleave other substrates however, the functional relevance of these substrates remains unclear. For example, caspase-1 may contribute to cell demise by cleaving many structural proteins (e.g. vimentin, actin, gelsolin, IQGAP1 and others (Table 3)). Caspase-1 may also regulate RNA-mediated processes and metabolism by cleaving ribonucleoproteins [87,89] and glycolytic enzymes [88] (Table 3)).

Known caspase-4 and -5 substrates are minimal, and efforts to identify them thus far have been limited. Outside the substrates mentioned above, caspase-5 cleaves the transcription factor Max after glutamic acid [110], and SYAP1 at an unknown site [89].

### Caspases and their substrates during evolution

Numerous caspase substrates are conserved throughout evolution. Inflammatory caspases are present in vertebrates, from the zebrafish [120,121] to higher primates [20] (Table 3; D (*Danio rerio*), M (*Mus musculus*), H (*Homo sapiens*)). Human inflammatory caspases share high similarities with higher primates caspases.

Multiple substrates seem to be conserved during evolution and many of their cleavage site position (Table 3) is highly conserved, suggesting a role for various caspases substrates throughout evolution. Ancestral reconstitutions of caspases support the co-evolution of caspases and their substrates [71,122].

## Caspase inhibitors

Whereas apoptotic caspase activity is controlled by the inhibitors of apoptosis proteins (IAP) [123], endogenous inhibitors controlling inflammatory caspase activity are poorly characterised.

SerpinB1 was suggested to be an endogenous inhibitor of inflammatory caspases in some cell types, such as neutrophils [124].

The CARD-only proteins (COPs) and PYD-only proteins (POPs) can inhibit inflammatory caspases indirectly by regulating inflammasome formation and/or caspase recruitment to their activating platform [125].

The involvement of inflammatory caspases in a range of diseases containing an inflammatory component (from obesity to cancer and sepsis [126,127]) supports the urgency to develop specific inhibitors that can target one or multiple inflammatory caspases.

Vertex developed a caspase-1/4 inhibitor, VX765, that was taken to clinical trials [128]. However, the studies were halted in stage 2 due to liver toxicity [129].

The development of caspase inhibitors that target exosites highlights the possibility of more specific inhibitors.

In addition, the development of exosite inhibitors has the potential to generate inhibitors that target deleterious functions of caspases, without affecting the beneficial ones, by modulating the cleavage of selected substrates.

## Perspectives

- Importance in the field: Inflammatory caspases are crucial for regulated immune responses and linked to diverse pathologies from sepsis to cancer.
- Summary of the current thinking: Inflammatory caspases are activated either by canonical or non-canonical inflammasomes. GBPs are novel innate immune sensors that form a non-canonical inflammasome and facilitate LPS presentation to caspase-4 and -11. Caspases recognise their substrates through substrate binding-pockets and use exosites to increase substrate selectivity.
- Future directions: Research to identify and characterise novel caspase substrates will expand our understanding of inflammatory caspases in health and disease. Future research will address how inflammatory caspases activity is controlled by endogenous mechanisms and inhibitors. Targeting caspase exosites may allow for the development of more specific pharmacological inhibitors.

## Open Access

Open access for this article was enabled by the participation of University of York in an all-inclusive Read & Publish pilot with Portland Press and the Biochemical Society under a transformative agreement with JISC.

## Abbreviations

ASC: apoptosis-associated speck-like protein
CARD: Caspase recruitment and activation domain
COP: CARD-only protein
DAMP: danger-associated molecular patterns
IDL: interdomain linker
LTA: lipoteichoic acid
PAMP: pathogen-associated molecular patterns
POP: PYD-only protein
PRR: pattern recognition receptor
PYD: pyrin domain
RDL: recruitment domain linker
